# Multi-Beam Conformal Array Antenna Based on Highly Conductive Graphene Films for 5G Micro Base Station Applications

**DOI:** 10.3390/s22249681

**Published:** 2022-12-10

**Authors:** Bin Zheng, Xiangyang Li, Xin Rao, Na Li

**Affiliations:** The Key Laboratory of Electronic Equipment Structure Design, Ministry of Education, Xidian University, Xi’an 710071, China

**Keywords:** flexible antenna, highly conductive graphene film, multi-beam, MMW communication

## Abstract

Recently, micro base station antennas have begun to play a more important role in 5G wireless communication, with the rapid development of modern smart medical care, the Internet of things, and portable electronic devices. Meanwhile, in response to the global commitment to long-term carbon neutrality, graphene film has received significant attention in the field of antennas due to its low carbon environmental impact and high electrical conductivity properties. In this work, a conformal array antenna based on highly conductive graphene films (CGF) is proposed for 5G millimeter-wave (MMW) applications. The proposed antenna consists of three antenna arrays, with eight patch elements in each array, operating at 24 GHz, with linear polarization. Each antenna array’s current amplitude distribution coefficient is constructed by synthesizing a series-feeding linear array using the Chebyshev method. The measurement results demonstrated that the proposed CGF antenna exhibits a peak realized gain higher than 8 dBi in the bandwidth of 23.0–24.7 GHz. The proposed antenna achieves three independent beams from bore-sight to ±37° in conformal installations, with a cylinder radius of 30 mm, showing excellent beam-pointing performance. These characteristics indicate that the CGF can be used for the design of MMW micro base station antennas, fulfilling the requirements of the conformal carrier platform for a lightweight and compact antenna.

## 1. Introduction

The fifth-generation (5G) millimeter-wave (MMW) wireless communication system with the advantages of low latency, high transmission rate, high integration, and huge bandwidth has broad application prospects, including the areas of medical and health, intelligent transportation, virtual reality, industrial automation, and other fields [1,2] However, the 5G network requires many base stations, and the number of 5G base stations is 1.4∼2 times [3] greater than the number of 4G base stations due to their high power consumption and limited network coverage. Therefore, this may lead to more carbon emissions, which does not correspond with the global green communication development requirements [4].

The International Telecommunication Union has approved several MMW spectra for 5G applications, such as 24.25~27.5 GHz, 37~40 GHz, and 66~76 GHz [5,6,7]. References [8,9,10] show some research regarding traditional micro base station antennas, such as omnidirectional solid angle beam-switching micro base station antennas [8], wideband indoor micro base station antennas [9], polarization reconfigurable micro base station antennas [10], etc. However, these antennas use metal as the radiator. On the one hand, metal materials are not resistant to corrosion, making them difficult to recycle and degrade. On the other hand, metal exhibits poor flexural endurance and is hard to apply to a conformal carrier. Therefore, it is necessary to design an antenna for the micro base station whose radiation performance can be comparable to that of a metallic antenna, while exhibiting the advantages of environmental protection and flexibility.

Recently, flexible antennas based on novel conductive materials have been developed to solve these problems. Paper [11,12,13] introduces several new conductive materials, such as conductive polymers, metal nanoparticles, carbon nanomaterials, etc. In the study in reference [11], a reconfigurable antenna for 5G applications was fabricated using Galinstan microfluidics instead of a diode. However, the preparation process for microchannel technology is complicated. In Paper [12], 3D-printed antennas based on silver nanoparticles are proposed. However, antennas based on silver nanoparticles are expensive to manufacture; therefore, they are not suitable for mass production. Copper and other metal nanoparticles are easy to oxidize to form a layer of non-conductive oxides [13]. In contrast, graphene is well-received due to its excellent properties, such as extremely high sensitivity, light weight, environmental protection, corrosion resistance, and mechanical stability [14,15].

In addition to the lightweight, flexible, and environmental protection of graphene antennas, its raw material—graphite—is rich in energy storage and low in price, exhibiting broad application prospects. Graphene materials used in the antenna field have three main physical forms: graphene inks, nanographene particles, and multilayer graphene films. However, due to the low conductivity of the first two materials on a macro scale, graphene-based antennas [16,17] are often unsatisfactory. For graphene film, a single atomic plane graphene is a two-dimensional (2D) crystal, and the multi-atomic layer structure is considered a thin film of 3D materials [18]. For three or more layers, the spectra become increasingly complicated, as they have many peculiar properties [19]. The antenna based on multilayer graphene film has high conductivity and can solve the problem of low conductivity for graphene inks and particles. Several flexible antennas based on highly conductive graphene film (CGF) have been developed at low frequencies [20,21,22,23]. However, there are few applications in the MMW spectrum at present, and more intense efforts are in progress to explore the applications of CGF antennas for 5G communication. Specifically, reference [24] presented an MMW antenna array with 32 elements based on high conductivity graphene assembled film (GAF) for 5G mobile communication. Paper [25] proposed two-beam scanning phased array antennas based on highly conductive GAF for 5G mobile communication applications. However, these are planar-integrated antennas. In contrast, Paper [26] studied the influence of the radius of a cylindrical supporting structure on the radiation properties of a conformal MMW antenna array, but its radiant surface is the traditional metal copper. To date, there has been little research on MMW conformal CGF antennas for non-planar integration.

To explore the application of graphene film in realizing flexible conformal MMW communication, this work proposes a novel graphene multi-beam conformal array antenna fabricated by laser prototyping. To the best of our knowledge, this is the first time that graphene materials have been adopted to design conformal MMW multi-beam arrays for micro base station applications.

## 2. Materials and Methods

### 2.1. Characterization of the CGF

**Figure 1:**
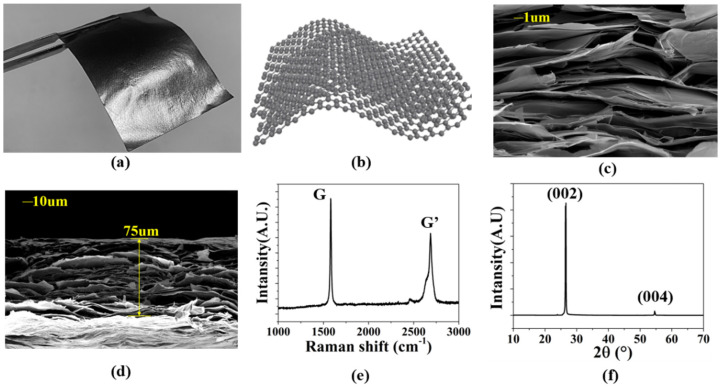
(**a**) The photograph of the CGF. (**b**) Molecular structure of CGF. (**c**) The SEM image of CGF. (**d**) The thickness of CGF. (**e**) Raman spectra of CGF. (**f**) XRD pattern of CGF.

In order to carry out the antenna design, we first analyze the material properties of the multilayer graphene. Figure 1a is a photograph of the CGF sample. The film surface is glossy, which means that the graphene sheet has good uniformity and density, as well as good flexibility [23]. Figure 1b exhibits the molecular structure of the graphene film, showing that graphene is a multilayer 2D honeycomb lattice structure, with tightly packed carbon atoms. Therefore, the lightweight CGF is easy to achieve. Meanwhile, the multilayer structure can be observed clearly in the film’s cross-section electron microscopy (SEM), shown in Figure 1c. Figure 1d exhibits the SEM photograph of CGF, and the thickness of CGF used in this experiment was 75 μm. Figure 1e shows the Raman spectrum of the CGF. The G-peak of the Raman scattering process is near 1587 cm^−1^, without an obvious D-peak, and the G-peak is significantly sharper than the G′, indicating that the multilayer graphene is different from the structure of graphite (G′ > G). Figure 1f shows the X-ray diffraction pattern of CGF. The characteristic peak (002) occurs at 26.46°, indicating that CGF has a graphene layer stacked structure. The diffraction peak (004) indicates that CGF has a certain degree of graphitization.

We have studied the structural properties of CGF from different aspects, as shown in Figure 1. However, for an antenna design, we are more concerned about the conductivity of the CGF material and its scattering mechanism. For graphene of a small micrometer size, the conductivity can be calculated using the Kubo formula [26,27]. When the frequency is lower than 8 THz, the graphene conductivity can be approximately expressed as [26,27]:

(1) $$\sigma_{intra}\left(\omega ,\mu_{c},\Gamma ,T\right)=-j\frac{e^{2}K_{B}T}{\pi \hbar^{2}\left(\omega -j2\Gamma \right)}[\frac{\mu_{c}}{K_{B}T}+2\ln\left(e^{-\frac{\mu_{c}}{K_{B}T}}+1\right)]$$

where *e* is the electron charge, $K_{B}$ is Boltzmann constant, Γ is the electron–phonon scattering rate, *ℏ* is Plank’s constant, *ω* is the frequency of the incident wave, $\mu_{c}\;$ is the chemical potential, and *T* is the temperature. 

**Figure 2:**
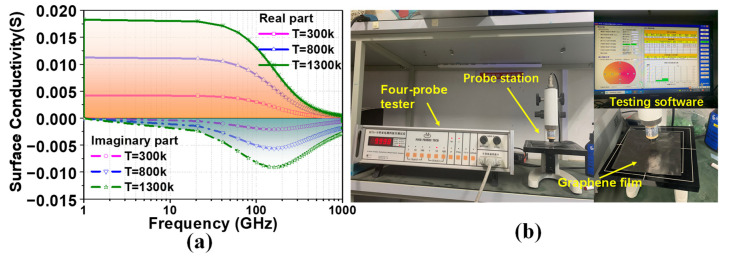
(**a**) Calculation and (**b**) measurement of the surface conductivity of the CGF.

For our work, we used the value $\mu_{c}\;$= 0 eV. Calculated using MATLAB software, the relationship between graphene surface conductivity and frequency at different temperatures is shown in Figure 2a. The results show that the surface conductivity of graphene does not change with the frequency in the microwave band, and the surface conductivity increases slightly with the increase in temperature. In addition, for graphene with more layers, reference [28] established a calculation model of the electron scattering of the mechanism of graphene films based on the Boltzmann transport equation and the 2D electron gas theory. The results show that the conductivity of micron-level multilayer graphene scarcely varies with the number of layers. However, these mathematical models of the electrical conductivity of multilayer graphene are an ideal conclusion, without considering the influence of particle doping and fabrication process errors.

To obtain the accurate conductivity of the CGF sample used in our work, we use a four-probe tester to measure its square resistance, as shown in Figure 2b. Through testing, it can be determined that the sheet resistance *R_s_* of the CGF material used in this paper is between 0.01~0.03 Ω/□, where the “Ω/□” is the unit of the sheet resistance of the graphene [24]. Based on this condition, the bulk conductivity *σ* of the CGF can be calculated according to the equation in [29]:

(2) $$R_{s}=\frac{1}{\sigma t}$$

where *σ* is bulk conductivity, and *t* is the sample thickness.

Thus, the conductivity of CGF with a thickness t of 75 μm is about 4.4 × 10^5^–1.33 × 10^6^ S/m. Although the conductivity value of the CGF (*σ* = 3.4 × 10^6^ S/m) is one order lower than those of other conventional solid metals (such as copper and aluminum), there is no change in the EM behavior, particularly at microwave frequencies. In summary, the CGF exhibits excellent electrical conductivity and has good application potential for antenna design.

### 2.2. Chebyshev Linear Series-Fed Antenna Array

In order to verify the application feasibility of the above CGF samples in practical engineering, we designed a broadband MMW linear series-fed array antenna and applied it to 5G micro base station communication. In the layout of today’s 5G micro base station antenna, the size should be as small as possible and the profile as low as possible, which will be more convenient for both installation and concealment. Therefore, most 5G micro base station antennas have the characteristic of a low profile. However, under the requirements of small size and low profile, the designed antenna often has a small gain and cannot meet the needs of daily life. Therefore, within a certain small size, determining how to maximize the use of volume in designing a broadband and high-gain antenna has become a major problem.

**Figure 3:**
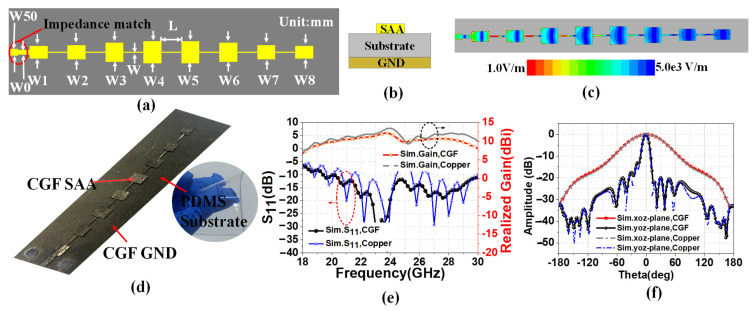
(**a**) Structure; (**b**) cross-section; (**c**) electric field distributions at 24 GHz; (**d**) physical picture of the proposed CGF antenna. Comparison of CGF and copper antennas in (**e**) reflection coefficients and peak gains; (**f**) xoz/yoz-plane radiation patterns.

The linear series-fed array antenna (SAA) has the advantages of low profile and high gain. In addition to this, wide bandwidth can be achieved by properly designing impedance matching. Thus, we proposed a linear series-fed array antenna made up of eight patch elements, satisfying the Chebyshev distribution. Figure 3a exhibits the structure of the proposed CGF flexible SAA. The array comprises a 50 Ω microstrip line, a quarter-wavelength impedance matcher, and eight patch elements. We used an impedance matcher to alter the characteristic impedance between the load and the antenna elements [25]. The design uses the Chebyshev synthesis method to calculate the current amplitude distribution coefficient of each array element. We took the physical sidelobe level (SLL) lower than 20 dB as the analysis limit threshold, and the current amplitude distribution of the CGF antenna is presented as follows [30]:

I1:I2:I3:I4:I5:I6:I7:I8 = 0.58:0.66:0.88:1:1:0.88:0.66:0.58(3)

where I4, I5 represents the normalized current amplitude value of the central array elements. 

The amplitude distribution of the excitation current of microstrip SAA is controlled by the element width gradient method [1]. In other words, the ratio of the widths of the elements Wn is equal to the current amplitude ratio In, as shown in Figure 3a. Figure 3b is the cross-section view schematic diagram of the proposed CGF antenna’s structure. Figure 3d shows the physical sample image of the laser-engraved graphene antenna. The polydimethylsiloxane (PDMS) (εr = 2.7, tan δ = 0.013, H = 0.5 mm) is used as a medium substrate for the proposed antenna because of its low degree of freedom and strong adhesion. The back of the dielectric substrate is the CGF ground, and the front consists of three identical CGF radiation patches. The conductivity of CGF is set as 1.13 × 10^6^ S/m, with a thickness of 0.075 mm.

In order to compare the radiation performance of graphene and traditional copper antennas, modeling analysis was carried out through high-frequency structure simulation (HFSS) software. Figure 3c demonstrates the electric field distribution of the CGF SAA at 24 GHz. Most of the electromagnetic energy of SAA is realized by the spatial distribution of W1–W4, while the spatial arrangement of W5–W8 will modify the impedance matching of the SAA. In addition, by adjusting the size of the rectangular patches and the RF line, the antenna structure is optimized to achieve broadband matching. Table 1 provides the optimized parameters of the variables of the proposed CGF antenna. The designed antenna works at 24 GHz. As shown in Figure 3e, the peak gain of graphene SAA from 23 GHz to 25 GHz was greater than 8 dBi, and the maximum gain was 10.23 dBi. In the range of 18–30 GHz, the simulated S_11_ is lower than −10 dB, indicating that the antenna possesses broadband performance. A copper antenna of the same size as the CGF antenna has higher resonant frequency. The realized gain of copper antenna is 0.5-1.5dBi higher than that of CGF antenna, which is due to the higher conductivity of copper (5.71 × 10^7^ S/m) than graphene (1.13 × 10^6^ S/m). As shown in Figure 3f, the simulated main-beam half-power bandwidths (HPBWs) are approximately 11.77° (−6.64° to 5.13°) and 66.89° (−33.50° to 33.39°) in the yoz-plane and xoz-plane, respectively. The copper antenna of the yoz-plane nearly coincides with that of the graphene antenna. The simulation results show that CGF and copper have similar radiation characteristics, and CGF can replace copper in antenna design.

## 3. Results

**Figure 4:**
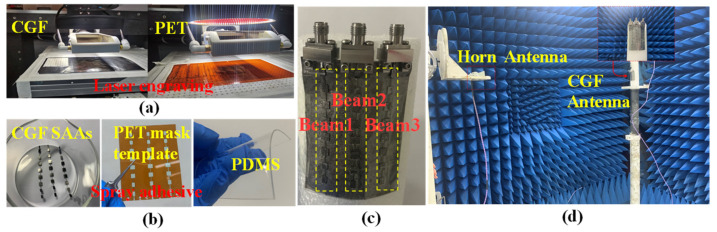
Fabrication process of the CGF antenna: (**a**) laser engraving and (**b**) photolithographic samples; (**c**) the prototype; (**d**) anechoic chamber measurement environment.

**Figure 5:**
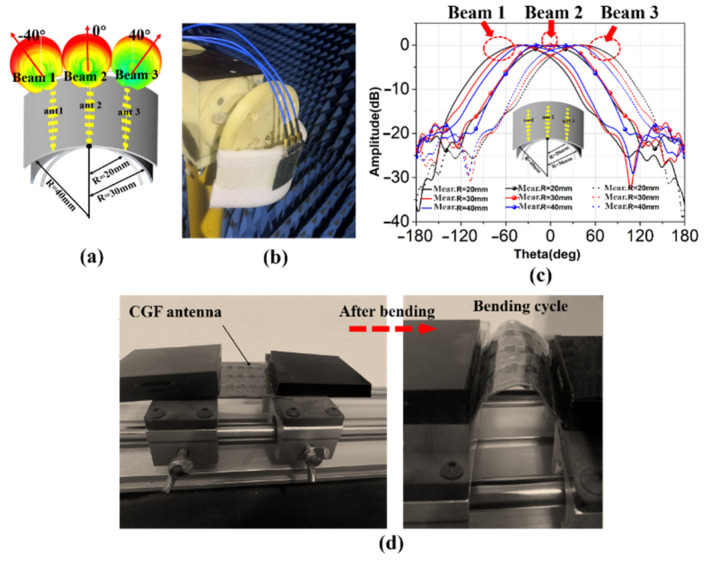
Measurement results: (**a**) conformal CGF antenna on the cylinder with different radii; (**b**) far-field measurement environment; (**c**) yoz-plane radiation patterns; (**d**) bending cycle experiments with the CGF antenna.

**Figure 6:**
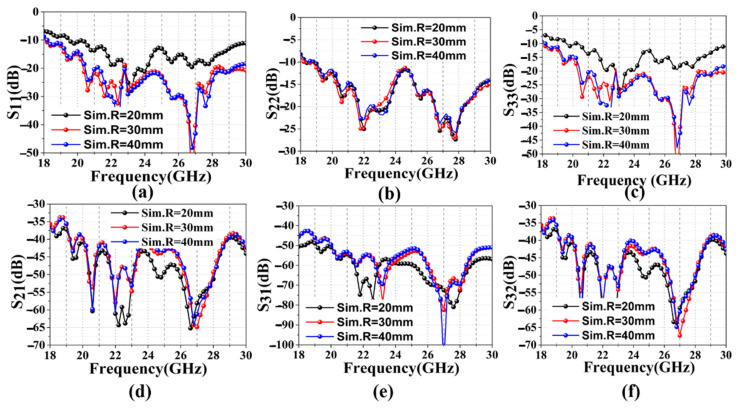
Measured S-parameter: (**a**) reflection coefficients of ant1; (**b**) reflection coefficients of ant2; (**c**) reflection coefficients of ant3; (**d**) mutual coupling S_21_; (**e**) mutual coupling S_31_; (**f**) mutual coupling S_32_.

**Figure 7:**
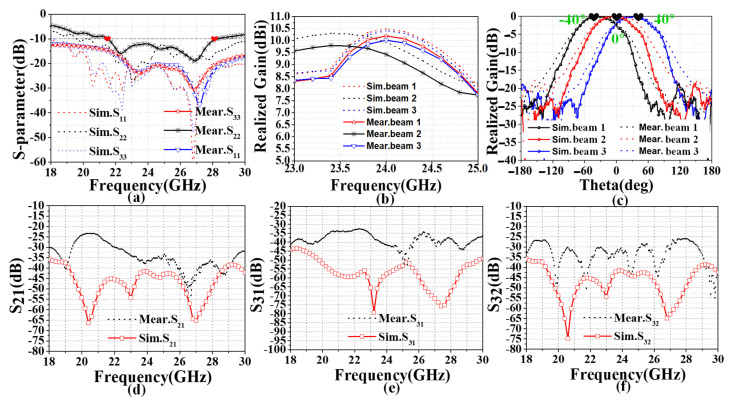
Normalized measured and simulated results for the CGF conformal antenna with the radii of 30 mm; (**a**) S-parameter of the CGF antenna; (**b**) realized gains; (**c**) the main lobe direction; (**d**) mutual coupling of ant1 and ant2; (**e**) mutual coupling of ant1 and ant3; (**f**) mutual coupling of ant2 and ant3.

In addition to the high gain characteristics mentioned above, with the rapid development of communication technology, 5G base station antennas mainly require a wider impedance bandwidth, a higher isolation (reducing coupling), a more stable radiation pattern, and more beam coverage. In order to meet the multi-beam and high-isolation characteristics of the micro base station, we placed three SAAs, as designed above, on one dielectric substrate to generate three beams in different directions; the spacing of the arrays was approximately 0.5 λg (the wavelength in the medium). For ease of description, the SAAs antennas are named ant1, ant2, and ant3, respectively, as shown in Figure 4. The detailed experimental process is as follows:

- Firstly, we fabricated a graphene antenna prototype.
- Then, to illustrate the influence of the conformal radius on the mutual coupling of the three linear arrays, we tested the S-parameter and the far field radiation patterns of the CGF antenna, as shown in Figure 5 and Figure 6. The antenna was conformed on foam cylinders with radii of 20 mm, 30 mm, and 40 mm, respectively.
- Finally, a 30 mm conformal cylinder was used to compare the measured and simulated reflection coefficients and the realized gains, as shown in Figure 7.

### 3.1. The Fabrication of the Conformal CGF Antenna

The proposed CGF antenna prototype was realized through a laser engraving process, and the cutting accuracy of the laser engraving machine is 20 μm, which can meet the processing accuracy requirements in MMW [31]. Figure 4a illustrates the schematic diagram of the fabrication process of the proposed CGF antenna. Firstly, an LPKF laser engraver cuts CGF to obtain patches, as well as a polyethylene terephthalate (PET) mask template, as exhibited in Figure 4b. Then, the PET mask template is placed on the PDMS substrate, sprayed with glue, and the CGF patch is pasted on it after the glue has dried. Finally, the CGF ground is pasted on the back of the PDMS in the same way. The PET is then remove to obtain the CGF antenna, and the CGF antenna is connected with three 50 Ω weld-free SMA connectors curved on a foam cylinder with a radius of 30 mm, as exhibited in Figure 4c. 

Specifically, the PDMS adopts Dow Corning’s Sylgard 184 silicone elastomer kit. First, liquid PDMS and a curing agent were mixed at a ratio of 10:1 by volume. Then the liquid mixture was poured into a mold and thermally cured at 70 °C for 2 h [32]. Finally, the cured PDMS can be obtained after peeling it off of the mold. The fabricated antenna has excellent flexibility, with a total thickness of 0.65 mm. The radiation patterns and S-parameters are measured in a far-field MMW anechoic chamber [26], as exhibited in Figure 4d.

### 3.2. Measured Results

#### 3.2.1. Conformal Experiment

Firstly, the antenna is conformed on a foam cylinder with radii of 20 mm, 30 mm, and 40 mm, respectively. For example, Figure 5a shows the 3-D radiation pattern with a radius of 30 mm, generating beams in three different directions. Figure 5b is the photograph of the conformal antenna tested in the anechoic chamber. Figure 5c illustrates the numerical scanning results of the xoz-plane, where the angles corresponding to the maximum gains of different radii are approximately ±42° (R = 20 mm), ±40° (R = 30 mm), and 37° (R = 40 mm), respectively. The results show that the beam scanning angle of the antenna increases with the decrease of the conformal radii.

In addition, since CGF is highly fragile, we had mechanical reliability experiments for the CGF antenna. Figure 5d shows the bending cycle experiments of the CGF antenna. Figure 5d focuses on the analysis of the bending cycle of the CGF antenna. The CGF can maintain unchanged resistivity after 50 bending cycles at a frequency of 0.5 Hz, while beginning to break after 50 cycles, due to the size of the microstrips being so tiny in the MMW band. However, the CGF antenna still exhibits good flexibility and mechanical stability, and 50 bending cycles can fully meet the conformal application requirements of micro base stations.

Then, to illustrate the influence of the conformal radius on the mutual coupling of the three linear arrays, we tested the S-parameter of the CGF antenna using a vector network analyzer (VNA), as shown in Figure 6. Figure 6a–c represent the reflection coefficients of ant1, 2, and 3, respectively, tested under different bending radii. Figure 6a,c shows that ant1 and 3 are greatly affected by the bending radius, and the changing trends remain the same. With the increase in the bending radius, the reflection coefficient of S_11_ and S_33_ decreases. It is also found that the reflection coefficient becomes less sensitive to the bending radius as it changes from 30 mm to 40 mm. As shown in Figure 6b, ant2 is the least sensitive to the bending radius, and S_22_ does not change significantly as the bending radius changes from 20 mm to 40 mm. As exhibited in Figure 6d–f, the mutual coupling between S_21_, S_31,_ and S_32_ is nearly below −40 dB, −50 dB, and −40 dB, respectively. The results show that the mutual coupling decreases with the increase in the conformal radii. The results indicate that the CGF antenna exhibits low mutual coupling and stable radiation properties under different bending radii.

#### 3.2.2. Comparison of the Simulation and Measurement

The measured reflection coefficients of the fabricated antenna were measured and compared using a vector network analyzer (VNA), as shown in Figure 7. As illustrated in Figure 7a, the measured S_11_, S_22_, S_33_ are lower than −10 dB in the range of 21.5 GHz–28 GHz, indicating that the antenna possesses broadband performance. Among these, the S_11_ and S_33_ move towards the high frequency due to the effect of bending in changing the current path. Figure 7b compares the measurement and simulation of the radiation patterns at 24 GHz. The peak realized gain of the CGF antenna from 23 GHz to 24.7 GHz was higher than 8 dBi, and the maximum gains in three directions were 10.23 dBi, 9.48 dBi, and 10.11 dBi, respectively. The measurements agree well with the simulations in the xoz-plane. Figure 7c illustrates the numerical scanning results of the xoz-plane. To describe the beam orientation more clearly, we adopted the normalized directional graph gain to describe the beam orientation. The three SAAs in a conformal arrangement on a cylinder with a 30 mm radius provided beam scanning from −40° to +40°.

The S_21_ and S_32_ have similar values below −20 dB over the frequency range from 18 to 30 GHz, and the S_31_ is below −30 dB, as shown in Figure 7d,e. Figure 7f shows that ant2 and 3 have a lower isolation below −20 dB, due to the greater distance between the elements. Thus, it can be seen that the actual measured structure of the S-parameter is slightly higher than the simulation result. On the one hand, this discrepancy is due to the slight effect of the supporting foam. However, the coupling effect between the antenna elements is more sensitive at high frequencies. The characteristic of the coupling effect leads to the element excitation amplitude error and phase inconsistency, which affects the antenna’s overall radiation performance. However, the effects are slight, and the results show that the proposed CGF antenna arrays still have good isolation characteristics.

Table 2 compares gain values and the main lobe direction between the simulated and measured values. In the conformal installation, for the simulated and measured gain values of ant2, the difference is 0.5 dBi. For ant1 and ant3, differences of 0.66 dBi and 0.71 dBi occurred between the simulated and measured results. The maximum difference in the direction of the main lobe between the simulation and the measurements is approximately 3°. 

## 4. Discussion

To clearly illustrate the focus and innovation of this work, Table 3 compares the recently published results for high-conductivity graphene antennas with those from our work. As can be seen, Paper [13,18,19,20,21] designed several graphene antennas working at the low-frequency band, which are mainly suitable for wearable applications. However, these antennas have low gain and narrow bandwidth. Specifically, reference [22] presented an MMW antenna array with 32 elements, based on high conductivity graphene assembled film (GAF) for 5G mobile communication. Paper [23] proposed two beam-scanning phased array antennas based on the highly conductive GAF for 5G mobile communication applications. However, these are planar integrated antennas. To date, there has been little research on the MMW conformal CGF antennas for non-planar integration. Therefore, we proposed a multi-beam CGF antenna for MMW micro base stations, with high gain and broad bandwidth characteristics.

## 5. Conclusions

In this work, a multi-beam conformal array antenna made of flexible CGF materials was successfully designed. The measured results were in good agreement with the simulations, demonstrating that a peak realized gain greater than 8 dBi in the bandwidth of 23.0–24.7 GHz and three independent beams from bore-sight to ±37° were achieved. The experimental results show that CGF and copper possess similar radiation characteristics. In addition, the proposed CGF array antenna offers good flexibility, corrosion resistance, light weight, and environmental protection performance, which are all superior to traditional metallic antennas. For future work, this CGF array antenna will be studied further to provide more directive beams and to integrate with the beam-switched feed system. Thus, the proposed CGF antenna has great significance in the development of wireless communication for conformal devices, especially in MMW applications.

## Figures and Tables

**Table 1:** Geometrical parameters of the CGF SAA.

Parameter	*L*	*W*	*W*1	*W*2	*W*3	*W*4	*W*5	*W*6	*W*7	*W*8	*W*0	*L*0	*W*50	*L*50
Value (mm)	3.60	0.33	2.44	2.78	3.71	4.20	4.20	3.71	2.78	2.44	1	2.14	1.34	1.5

**Table 2:** Comparison of simulated and measured gain and beams.

CGF Antenna	Simulated Gain (@24 GHz) (dBi)	Measured Gain (@24 GHz) (dBi)	Simulated Lobe Direction	Measured Lobe Direction
Ant1	10.89	10.23	−40°	−37°
Ant2	9.98	9.48	0°	0°
Ant3	10.82	10.11	40°	37°

**Table 3:** Comparison of recently published antennas based on graphene films.

Ref.	Antenna	Operating Frequency (GHz)	Bandwidth S11 < −10 dB (GHz)	Peak Gain (dBi)	Applications	Conformal
[15]	Single patch	3.51	5.7%	6.77	5G mobile communication	No
[20]	Single patch	7.45	60.16%	3.9	Wearable applications	Yes
[21]	CPW patch	4	34.2%	-	Wearable applications	Yes
[22]	Single patch	1.63	6.1%	-	Wearable applications	Yes
[23]	Single patch	5.8	13.8%	6.1	Wearable applications	Yes
[24]	4 × 8 Linear array	30	6.7%	15.07	MMW communication	No
[25]	1 × 10 Linear array	26	1.9%	15.75	5G applications	No
Our work	1 × 8 Linear array	24	26.3%	10.23	5G Micro Base Station	Yes

## Data Availability

Not applicable.
